# A new way of producing pediocin in *Pediococcus acidilactici* through intracellular stimulation by internalized inulin nanoparticles

**DOI:** 10.1038/s41598-018-24227-z

**Published:** 2018-04-12

**Authors:** Whee-Soo Kim, Jun-Yeong Lee, Bijay Singh, Sushila Maharjan, Liang Hong, Sang-Mok Lee, Lian-Hua Cui, Ki-June Lee, GiRak Kim, Cheol-Heui Yun, Sang-Kee Kang, Yun-Jaie Choi, Chong-Su Cho

**Affiliations:** 1 Department of Agricultural Biotechnology, Seoul National University, Seoul, 08826 Republic of Korea; 2 Research Institute of Agriculture and Life Sciences, Seoul National University, Seoul, 08826 Republic of Korea; 3 Research Institute for Bioscience and Biotechnology, Kathmandu, 44600 Nepal; 4 Institute of Green-Bio Science & Technology, Seoul National University, Pyeongchang, Gangwon-do, 25354 Republic of Korea; 5 Co-Innovation Center of Beef Cattle Science and Industry Technology, Yanbian University, Yanji, Jilin, 133002 P. R. China

## Abstract

One of the most challenging aspects of probiotics as a replacement for antibiotics is to enhance their antimicrobial activity against pathogens. Given that prebiotics stimulate the growth and/or activity of probiotics, we developed phthalyl inulin nanoparticles (PINs) as prebiotics and observed their effects on the cellular and antimicrobial activities of *Pediococcus acidilactici* (PA). First, we assessed the internalization of PINs into PA. The internalization of PINs was largely regulated by glucose transporters in PA, and the process was energy-dependent. Once internalized, PINs induced PA to produce substantial amounts of antimicrobial peptide (pediocin), which is effective against both Gram-positive (*Salmonella* Gallinarum) and Gram-negative (*Listeria monocytogenes*) pathogens. When treated with small-sized PINs, PA witnessed a nine-fold increase in antimicrobial activity. The rise in pediocin activity in PA treated with PINs was accompanied by enhanced expression of stress response genes (*groEL*, *groES*, *dnaK*) and pediocin biosynthesis genes (*pedA*, *pedD*). Although the mechanism is not clear, it appears that the internalization of PINs by PA causes mild stress to activate the PA defense system, leading to increased production of pediocin. Overall, we identified a prebiotic in nanoparticle form for intracellular stimulation of probiotics, demonstrating a new avenue for the biological production of antimicrobial peptides.

## Introduction

Prebiotics are generally defined as non-digestible materials that stimulate the growth and/or activity of probiotics and/or other microorganisms in the gastrointestinal (GI) tract to confer favorable health effects on the host^1^. Basically, the health benefits are largely mediated by short chain fatty acids (SCFAs) produced by gut microbiota through the metabolism of prebiotics^2^. In general, dietary fibers consisting of carbohydrates are widely used as prebiotics because the fermentation of carbohydrate generates SCFAs. Interestingly, SCFAs such as acetate or propionate are selectively produced by lactic acid bacteria (LAB) of the *Lactobacillus* and *Bifidobacterium* genera, while butyrate is produced by *Clostridium* and *Eubacterium* genera^2^. Moreover, prebiotics alter the gut environment, such as the pH, viscosity, gut transit and interactions with other food components. Although a variety of compounds have been used as prebiotics, the most frequently used and studied dietary fiber is inulin. Inulin is naturally present as a polysaccharide in many plants, although the main industrial source is chicory root. Typically, inulin is a fructan derivative consisting of fructosyl residues (n = 2‒60) linked by β (2 → 1) bonds and a glucosyl residue as an end group. Due to the β (2 → 1) linkages in inulin, it is not digested by pancreatic enzymes in the upper GI tract. However, inulin is fermented by the microbiota present in the colon to produce SCFAs, leading to selective stimulation of the growth of specific bacterial populations in the host intestine and subsequently altering the host immune system.

Over the last few decades, there has been growing interest in the use of probiotics as potential alternatives for synthetic antibiotics and anti-inflammatory drugs, due not only to the side effects of synthetic drugs but also to the improper use of antibiotics promotes the development of antibiotic-resistant bacteria. Generally, the effects of probiotics can be classified into three modes of action for use as alternatives to antibiotics: 1) probiotics have a direct effect on other microorganisms or pathogens by producing antimicrobial substances such as bacteriocins, 2) probiotics can modulate host defenses including the innate and acquired immune system and 3) probiotics affect microbial products such as toxins and host products^3^. However, the use of probiotics as a replacement for antibiotics is challenging due to their low production of antimicrobial substances.

Hence, a number of strategies, including biological, physical, and chemical methods, have emerged to enhance the production of bacteriocins from probiotics. Although biological engineering strategies may propel probiotics to yield antimicrobial peptides with greater stability and enhanced features^4^, the biological processes are much more complicated, and there is a rising concern regarding genetically modified products among many consumers. Among physical methods, optimization by varying parameters such as pH, temperature and incubation period for bacteriocin production in *Lactobacillus*^5^ and *Pediococcus*^6^ was evaluated, but without taking into account the expression of other metabolites. In contrast, high hydrostatic pressure treatment in *Enterococcus* strains^7^ and *Weissella viridescens*^8^ was shown to enhance the production of bacteriocin with an antimicrobial effect against *Listeria monocytogenes*. Although several carbohydrates have been adopted as part of a chemical method to enhance the health benefits of probiotics, a detailed analysis of the effects of carbohydrate treatment on probiotics has not yet been performed. Moreover, there has been no exploration of the alteration of metabolite production in probiotics using carbohydrate nanoparticles.

Polymeric nanoparticles have been widely used in biomedical applications because they can deliver chemotherapeutics, proteins, genes and contrast agents as cargoes to the desired place of action or in response to specific biological or external stimuli^9^. In particular, they can be used to overcome cellular barriers for the delivery of hydrophobic drugs and macromolecules inside cells as polymeric nanoparticles that are internalized with cellular membranes into vesicles during endocytosis^10^. Most importantly, polymeric nanoparticles are easily formed by the self-assembly of hydrophilic polymers modified with hydrophobic groups due to the hydrophobic interaction of hydrophobic groups in the inner cores of the polymeric nanoparticles. For instance, hydrophobic groups such as phthalates can be incorporated into a water-soluble polymer (inulin) to form inulin nanoparticles through hydrophobic interactions of the phthalates.

In this study, we synthesized a number of phthalyl inulin nanoparticles (PINs) to develop them as prebiotics. We treated *Pediococcus acidilactici* (PA), a probiotic that produces pediocin (antimicrobial peptide), with each PIN separately and analyzed the changes in the antimicrobial activity of PA in response to each PIN by antimicrobial assays. We further explored the consequences of PINs on PA at the genetic level by transcriptome analysis. To the best of our knowledge, this is the first report to demonstrate improved antimicrobial activity of probiotics using a prebiotic in nanoparticle form.

## Results

### Synthesis and characterization of PINs

**Figure 1:**
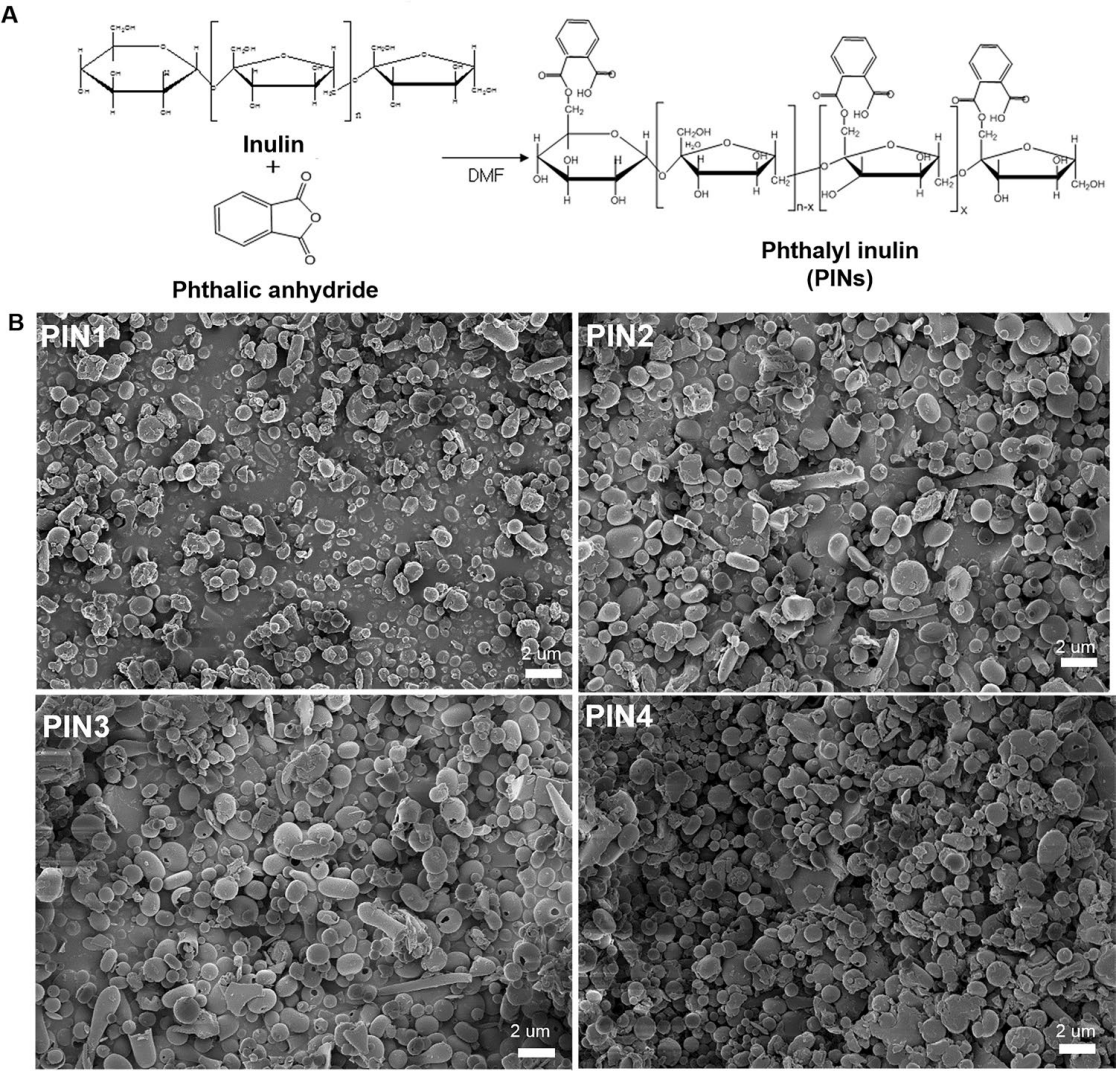
Chemical reaction scheme for the synthesis of PINs (**A**). For the reaction in each PIN synthesis, the molar ratio between phthalic anhydride and inulin was as follows: 0.3:1 (PIN1), 0.6:1 (PIN2), 1.2:1 (PIN3) and 2:1 (PIN4). Morphology of PINs observed by SEM (**B**). Magnification: 10,000×; Scale bar = 2 µm. (PINs: phthalyl inulin nanoparticles, SEM: scanning electron microscope).

The reaction scheme of the synthesis of PINs is shown in Fig. 1A. The degree of substitution of phthalate moieties in inulin was controlled by varying the molar ratio of phthalic anhydride to inulin, such as 0.3:1 (PIN1), 0.6:1 (PIN2), 1.2:1 (PIN3) and 2:1 (PIN4). The degree of substitution of phthalic groups in PINs was confirmed by ^1^H-NMR spectroscopy (Supplementary Fig. 1A). The peak assigned to the protons of phthalic acid appeared at 7.4–7.7 ppm, and the peak assigned to the protons of inulin appeared at 3.8 ppm in the NMR spectra. Based on the integration of protons in phthalic acid and protons in inulin, the PINs were named as follows: PIN1 (content of phthalic acid: 9.9 mol.-%), PIN2 (content of phthalic acid: 15.2 mol.-%), PIN3 (content of phthalic acid: 20.4 mol.-%) and PIN4 (content of phthalic acid: 27.4 mol.-%). The morphologies of the PINs were spherical with nanometer sizes when observed using a scanning electron microscope (SEM), and the number of smaller nanoparticles was greater in PIN4 as a consequence of the higher content of phthalic acid groups (Fig. 1B). The sizes of the nanoparticles measured by DLS were 365, 330, 320 and 224 nm for PIN1, PIN2, PIN3 and PIN4, respectively, signifying that the particle sizes of PINs decreased in the following order (PIN1 > PIN2 > PIN3 > PIN4) with an increase in conjugated phthalic acid groups in PINs (Supplementary Fig. 1B). Furthermore, the zeta-potentials of PINs measured by ELS were −21.29, −27.91, −26.34 and −23.64 mV for PIN1, PIN2, PIN3 and PIN4, respectively (Supplementary Fig. 1C). The negative zeta potential arose due to the non-reacted carboxylic acids in the phthalic moieties of PINs that are deprotonated at pH 7 (distilled water).

### Internalization of PINs into probiotics

**Figure 2:**
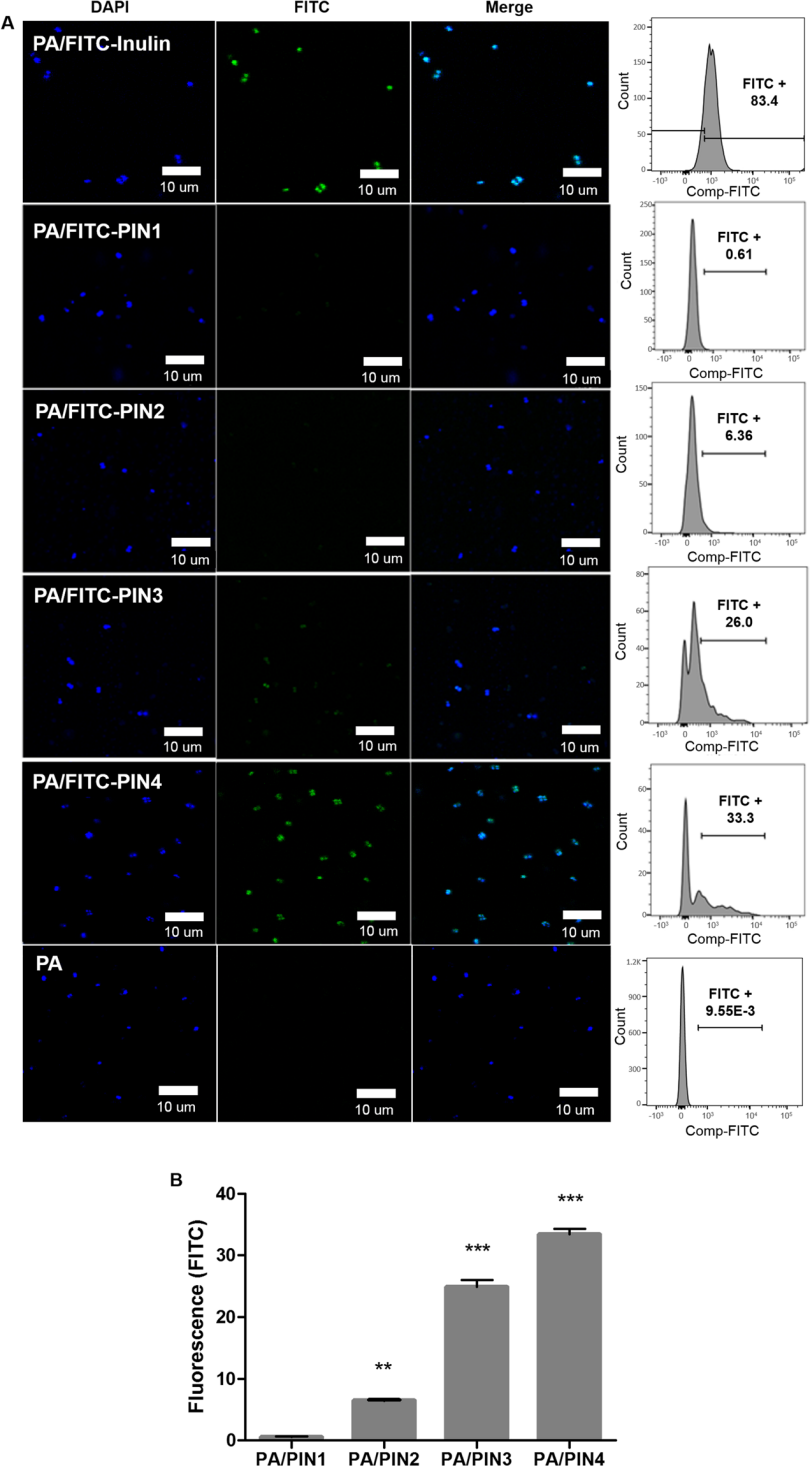
Analysis of the internalization of PINs in PA (**A**). Confocal images and FACS analysis were performed after treatment of PA with 0.1% (w/v) FITC-PINs or FITC-inulin for 3 min at room temperature. FITC-PINs or FITC-inulin are shown in green, and PA was stained blue with DAPI. The internalization of PINs after 3 min of treatment was quantified by FACS and statistically analyzed (**B**). Confocal and FACS data are representative of three independent experiments, and the average values are presented as the mean ± SEM of three independent FACS experiments by a bar chart. Statistical significance was analyzed between PA/PIN1 and other groups by one-way ANOVA, Tukey t test (*p < 0.05; **p < 0.01, ***p < 0.001). Scale bar = 10 µm. (PA: *Pediococcus acidilactici*, PIN: phthalyl inulin nanoparticle, CLSM: confocal laser scanning microscopy, FACS: fluorescence-activated cell sorting, FITC: fluorescein isothiocyanate, DAPI: 4’,6-diamidino-2-phenylindole).

To study the internalization of PINs into probiotics, PINs were conjugated to fluorescence isothiocyanate (FITC), and *Pediococcus acidilactici* (PA) was used as a probiotic strain. The internalization of FITC-PINs into PA was analyzed by confocal laser scanning microscopy (CLSM) and quantified by fluorescence-activated cell sorting (FACS). Initially, we observed in CLSM images that FITC-inulin was able to enter into PA within 3 min of incubation at room temperature (Fig. 2A). Similarly, internalization of PIN1, PIN2, PIN3 and PIN4 into PA was observed within 3 min of incubation at room temperature. Among the PINs, the highest internalization rate was observed for PIN4 (33.3%), while the internalization of PIN1 was only 0.61% (Fig. 2A,B). These results demonstrated that the internalization of PINs into PA increased with the decrease in particle sizes of PINs, suggesting a size-dependent internalization of PINs. Hence, unless otherwise stated, further experiments were selectively performed by treating PA with PIN4 only.

To further examine whether the PINs were on the surface or inside PA, PA were treated with FITC-PIN4, and CLSM was performed in Z-section mode. As shown in Supplementary Fig. 2A, the fluorescence intensity was highest at the center of PA, indicating the internalization of PIN4 into PA. In an alternative method to observe the internalization of PIN4 into PA, we used PIN4/curcumin to treat PA and observed the uptake by CLSM performed in Z-section mode. The result also showed that PIN4/curcumin were embedded in PA (Supplementary Fig. 2B).

To observe the morphological differences in PA due to the internalization of PINs, PA treated with or without PINs was observed using an energy-filtered transmission electron microscope (TEM). TEM images could not distinctly locate the PINs inside the PA (Supplementary Fig. 3). In fact, the internal compartments of PA appeared dark in the TEM images due to the thickness of the PA. Moreover, we observed the morphology of the PA treated with or without PIN4 by SEM to evaluate any structural changes in PA. The images demonstrated that PIN4-treated PA was indistinguishable from untreated PA (Supplementary Fig. 4).

**Figure 3:**
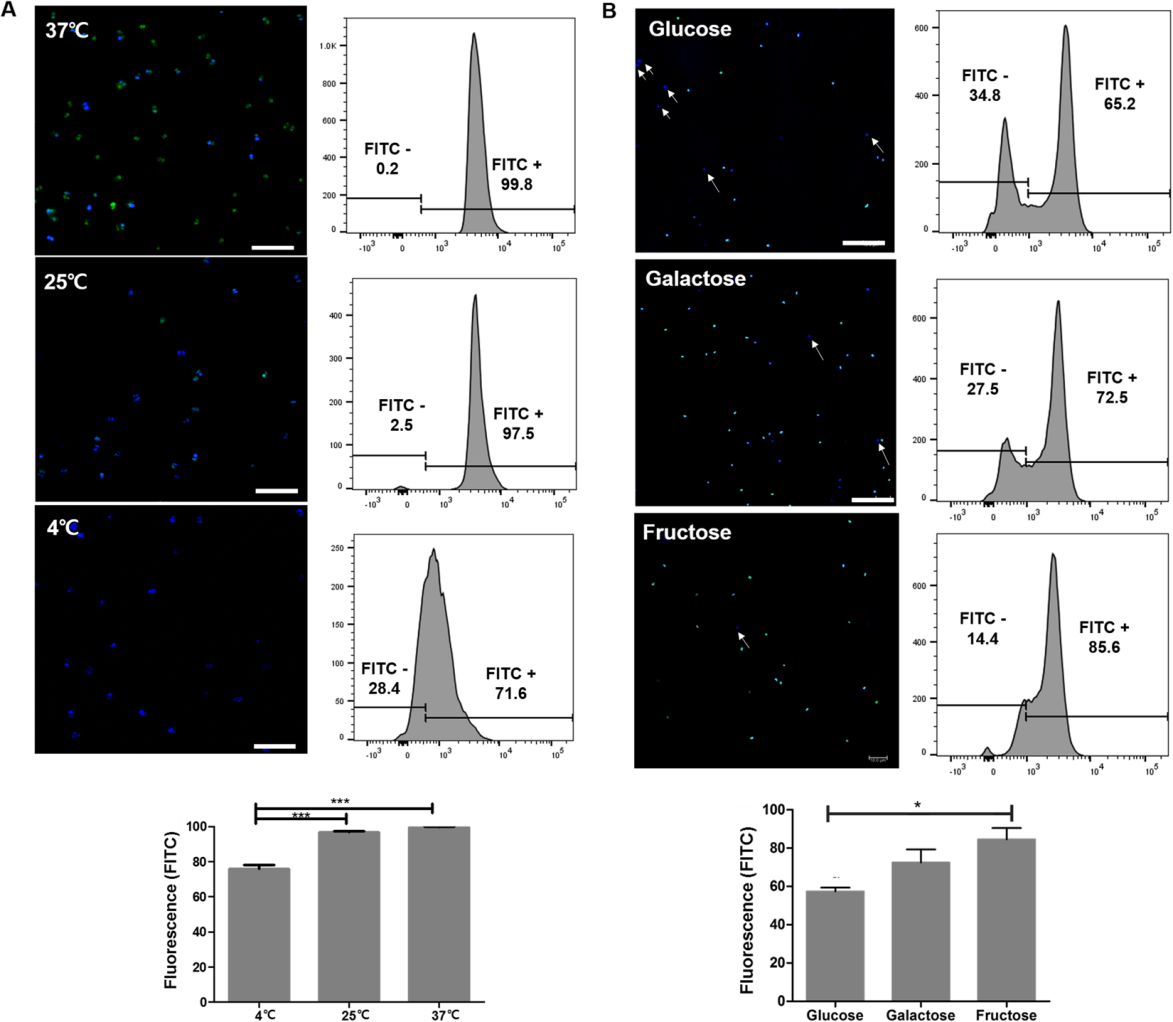
Analysis of the internalization of PINs in PA depending on temperature (**A**) and transporters (**B**). PA was treated with 0.1% (w/v) FITC-PIN4 at different temperature (4, 25, or 37 °C) for 6 h, and internalization was observed by CLSM and FACS (**A**). Next, PA, pre-incubated with 10% (w/v) glucose, fructose or galactose, was treated with 0.1% (w/v) FITC-PIN4 for 6 h at 37 °C, and the internalization was observed by CLSM and FACS (**B**). FITC-PIN4 is shown in green, and PA was stained blue with DAPI. The arrow point indicates a single DAPI signal. Confocal and FACS data are representative of three independent experiments, and the average values are presented as the mean ± SEM of three independent FACS experiments by a bar chart. Statistical significance were analyzed among the 4 °C, 25 °C and 37 °C groups and among the glucose, fructose and galactose groups by one-way ANOVA, Tukey t test (*p < 0.05; **p < 0.01, ***p < 0.001). Scale bar = 10 µm. (PA: *Pediococcus acidilactici*, PIN: phthalyl inulin nanoparticle, CLSM: confocal laser scanning microscopy, FACS: fluorescence-activated cell sorting, FITC: fluorescein isothiocyanate, DAPI: 4’,6-diamidino-2-phenylindole).

Further studies were performed to examine the internalization of PINs into PA according to the incubation temperature and transporters in PA. To check the temperature-dependent internalization of PINs into PA, FITC-PIN4 was treated with PA at 4, 25 or 37 °C for 6 h, and subsequently analyzed by CLSM and FACS (Fig. 3A). The results showed that the internalization of PIN4 into PA was significantly higher at 37 °C than 4 °C, suggesting an energy-dependent internalization of PINs. The results also indicated that PIN4 was more able to be internalized by PA at microbial growth than other temperatures.

Furthermore, a study was conducted to assess whether PINs were specifically internalized by transporter-mediated internalization. Typically, probiotics, pre-incubated with 10% (w/v) glucose, fructose or galactose, was treated with 0.1% (w/v) FITC-PIN4 for 6 h, and internalization was observed by CLSM and FACS. The results showed that the internalization of PINs was variably dependent on the specific transporter (glucose, fructose and galactose) (Fig. 3B). Pre-treatment with glucose impeded approximately 42.6% of PIN4 internalization, whereas pretreatment with galactose and fructose impeded 27.6% and 15.5% of PIN4 internalization, respectively. Overall, PIN internalization was significantly more retarded in the presence of glucose compared with fructose, suggesting that glucose transporters play a substantial role in PIN internalization.

### Effects of PINs on antimicrobial peptide production by PA

**Figure 4:**
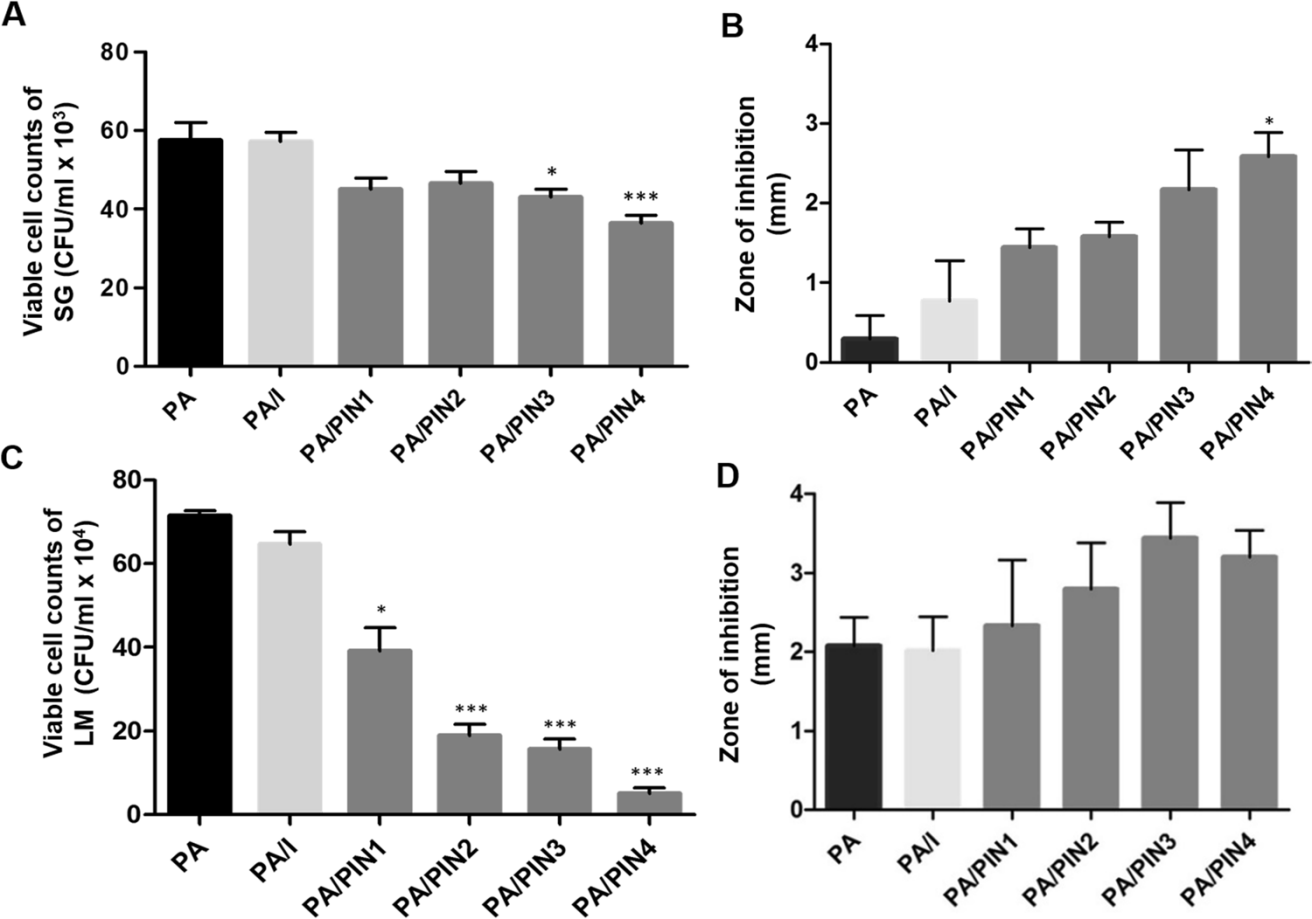
Antimicrobial efficacy of PA/PINs against SG and LM (**A**–**D**). PA treated with PINs or inulin were cultured with Gram-negative SG or Gram-positive LM, and the growth inhibition was calculated by CFU for SG (**A**) and LM (**C**). Similarly, the diameters of the growth inhibition of SG (**B**) and LM (**D**) on LB and BHI agar plates, respectively, were measured. Data are presented as the mean ± SEM of three independent experiments. Statistical significance was analyzed between PA and PA treated with PINs or inulin by one-way ANOVA, Tukey t test (*p < 0.05, **p < 0.01, ***p < 0.001). (PA: *Pediococcus acidilactici*, PIN: phthalyl inulin nanoparticle, I: Inulin, CFU: colony forming unit, PA: *Pediococcus acidilactici*, SG: *Salmonella* Gallinarum, LM: *Listeria monocytogenes*, LB: lysogeny broth, BHI: brain heart infusion).

To evaluate whether internalization of PINs by PA (PA/PINs) could affect the potency of antimicrobial peptide production, PA was separately treated with individual PINs, and the antibacterial potential induced by each PA/PINs was tested against Gram-negative *Salmonella* Gallinarum (SG) and Gram-positive *Listeria monocytogenes* (LM). Compared with PA alone, treatment with each PA/PINs resulted in higher antibacterial activity against both SG and LM in co-culture assays (Fig. 4A,C). Interestingly, the antibacterial potential of PA increased with a decrease in particle sizes of the internalized PINs. As the internalization of small-sized particles into PA was higher, it appeared that the increased antibacterial activity of PA was dependent on the amount of particles taken up by PA. Moreover, PA/PINs had relatively higher antibacterial activity than PA/I (soluble inulin) against both SG and LM. To examine if the enhanced antibacterial activity was induced by the PINs alone, SG or LM was treated with PINs in the absence of PA. Only the PINs themselves had no antibacterial properties (data not shown), indicating that the antibacterial potential emerged from PA due to interactions with internalized PINs. In addition, the antibacterial potential of PA in the presence of PINs was tested against SG and LM by agar diffusion test (Fig. 4B,D). The results of the agar diffusion test were evaluated by measuring the diameter of the zone of inhibition produced as a direct consequence of antimicrobial peptide production by PA/PINs. Consistent with the results of the antimicrobial test, the agar diffusion tests also showed similar pattern of antibacterial activity of PA/PINs against SG and LM. Again, the zone of inhibitions was relatively larger when the PA was internalized smaller PINs.

To determine an optimum concentration of PINs to induce the antibacterial potential of PA, different concentrations of PINs or inulin were applied to PA. While the treatment with 1.0% (w/v) PINs induced the antimicrobial potential of PA comparably higher than the treatment with 0.5% (w/v) PINs, there were no significant differences between the antimicrobial potentials of PA at these concentrations (Supplementary Fig. 5A). Hence, 0.5% (w/v) PINs was chosen to induce PA in the antimicrobial activity assay for further study. Accordingly, the probiotics were treated with different inulin nanoparticles (INs) prepared using different kinds of hydrophobic groups, such as acetyl or propyl instead of the phthalic group in inulin, to assess the specificity in the antibacterial properties of probiotics according to the nature of the hydrophobic group in INs. The probiotics internalized with the acetyl inulin nanoparticles (AIN) and propyl inulin nanoparticles (PrIN) also showed antimicrobial properties in probiotics (Supplementary Fig. 5B). The results indicated that the induction of antimicrobial property in probiotics was not specific to the nature of hydrophobic groups in INs.

### Effects of PINs on growth and pH changes of PA

**Figure 5:**
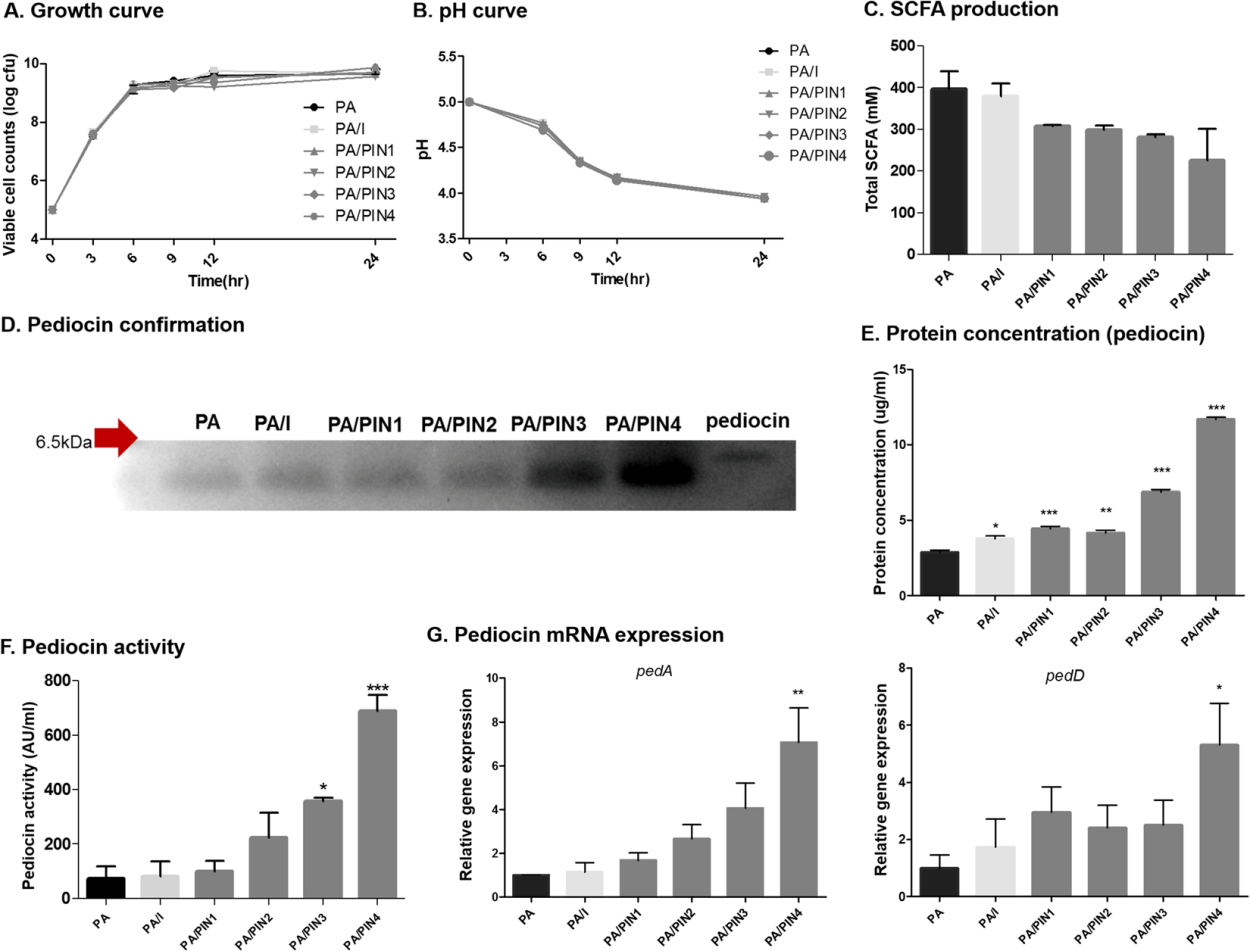
Analysis of the biological effect of PINs on PA. Measurement of the growth of PA (**A**) and pH of the culture medium (**B**) among PA groups with internalized PINs or inulin. Detection of the production of SCFAs (**C**). The molecular weight of pediocin was determined by SDS-PAGE using a reference pediocin. The full-length gels are presented in Supplementary Fig. 8 (**D**). Quantification of pediocin by the Bradford assay (**E**). Determination of the activity of pediocin (AU/ml) by measuring the diameter of the inhibition zone produced by PA (treated or untreated) against the growth of LM. (**F**). Relative mRNA expression of *pedA* and *pedD* compared with 16 S rRNA expression (**G**). Data are presented as the mean ± SEM of three independent experiments. Statistical significance was analyzed between PA and the other groups by one-way ANOVA, Tukey t test (*p < 0.05; **p < 0.01, ***p < 0.001) (PA: *Pediococcus acidilactici*, PIN: phthalyl inulin nanoparticle, I: inulin, SCFA: short chain fatty acid, AU: arbitrary unit, SDS PAGE: sodium dodecyl sulfate polyacrylamide gel electrophoresis).

To observe the changes in the growth conditions of PA after treatment with PINs or inulin, viable cells were counted at different time intervals (Fig. 5A). The results of the PA growth curve with or without PIN or inulin treatment showed no remarkable differences in PA growth. The pH of the culture medium of PA after treatment with PINs or inulin was also measured to evaluate the changes in lactic acid production (Fig. 5B). Consistent with the growth curve, the pH curve of the PA with or without treatment with PINs or inulin also showed no significant changes in the pH of the culture medium among the groups. The results indicated that internalization of PINs or inulin had no effect on the normal growth of PA.

### Effects of PINs on short chain fatty acid (SCFA) production by PA

To further examine any internal changes in PA by PINs, PA was treated with PINs or inulin, and commonly secreted products from PA such as SCFAs were analyzed. The results indicated that total SCFA contents in the culture medium of PA decreased after the treatment with PINs or inulin (Fig. 5C). The amount of total SCFA production in PA appeared to depend inversely on the particle sizes of internalized PINs. The greater the internalization of smaller PINs into PA, the less the production of SCFAs in PA. Although the production of SCFAs was comparably lower in PA by PINs than inulin, there were no significant differences among the treatment groups.

### Effects of PINs on pediocin production by PA

To determine the variations in the production of pediocin in PA by PINs, the pediocin from PA, PA/PINs and PA/I was isolated, confirmed and quantified by cell lysis, SDS-PAGE and the Bradford assay, respectively. First, we confirmed the molecular weight of isolated pediocin by SDS-PAGE using a standard pediocin as a reference (Fig. 5D). The results showed that the molecular weight of isolated pediocin was approximately 3.5 kDa. Additionally, SDS-PAGE showed that PA/PINs showed increased production of pediocin compared with the PA group under the same isolation conditions. Furthermore, the isolated pediocin from each PA with or without treatment was quantified by the Bradford assay. Compared with PA, PA/PINs and PA/I showed significantly higher production of pediocin (Fig. 5E). The results revealed that the production of pediocin was 4-fold higher in PA/PIN4 than PA alone. Similarly, the specific activity of the isolated pediocin was measured by the arbitrary unit. Consistent with the results obtained for pediocin production, PA/PINs and PA/I showed significantly higher pediocin activity than PA alone. Particularly, the activity of pediocin was significantly higher (9-fold) in PA/PIN4 compared with PA (Fig. 5F). Altogether, the production of pediocin in PA/PINs also increased with a decrease in the size of internalized PINs into PA.

To evaluate the variations of pediocin production in PA at the genetic level, a study was undertaken to compare the gene expression profiles of pediocin biosynthetic genes using quantitative real-time PCR (qRT-PCR) (Fig. 5G). Four pediocin genes (*pedA*, *B*, *C and D*) were selected, and 16 s rRNA were used for normalization. Following 24 h of treatment of PA with PINs or inulin, the relative gene expression of *pedA* was substantially higher in PA/PINs or PA/I than PA. Similarly, the expression level of *pedD* was variably higher in PA/PINs or PA/I than PA. In both cases, there were significant differences in the expression levels of *pedA* and *pedD* in PA/PIN4 compared with PA (Fig. 5G). However, the level of *pedC* expression showed no differences among groups, and the level of *pedB* expression was too low to detect by qRT-PCR (data not shown). These gene profile data clearly revealed the variations in the expression levels of pediocin biosynthetic genes in PA when internalized to PINs. In contrast, there was no significant difference in the production of hydrogen peroxide (H_2_O_2_) in PA/PINs compared with PA or PA/I (Supplementary Fig. 6).

### Effects of PINs on the transcriptome of PA

**Figure 6:**
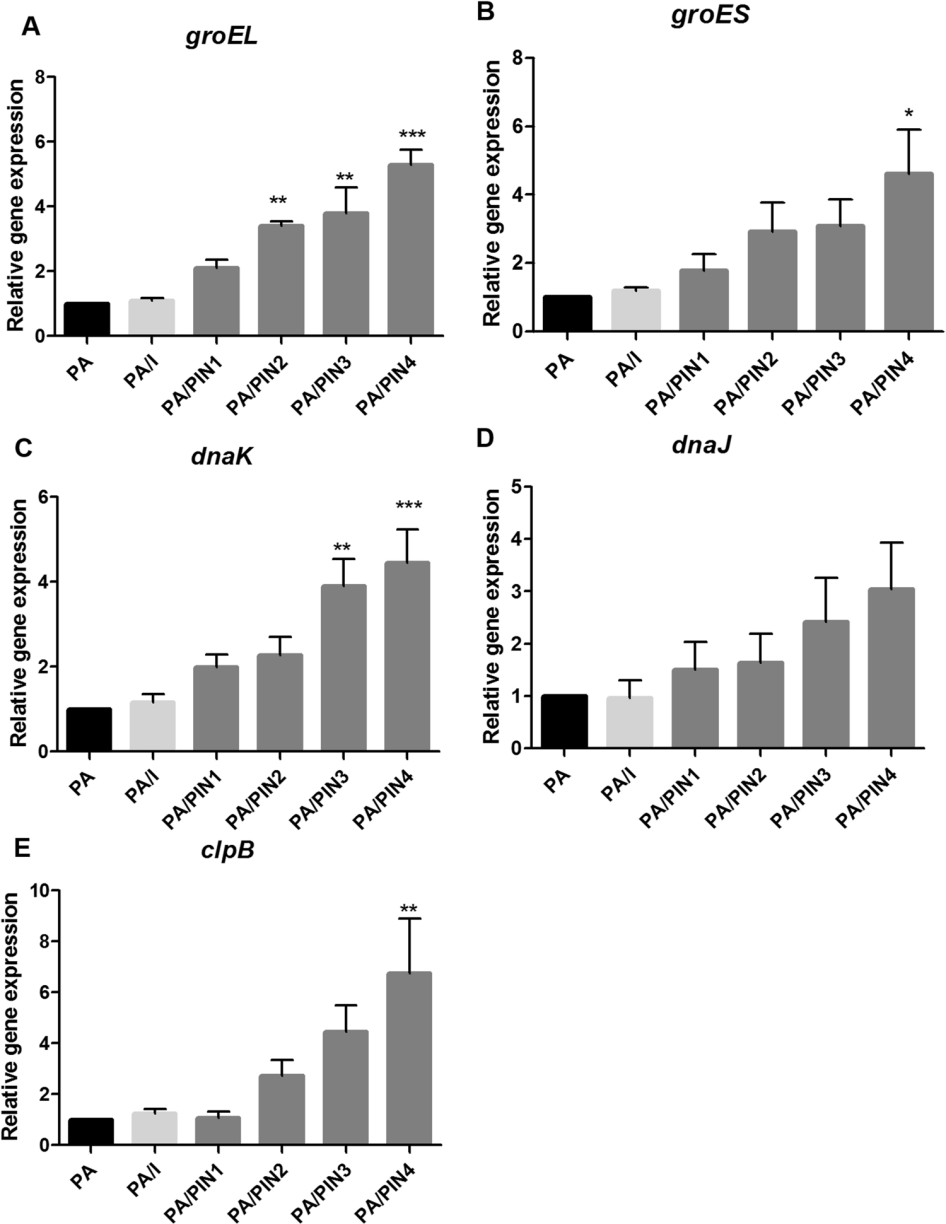
Analysis of gene expression related to the stress response in PA treated with PINs. The transcriptional expression of *groEL* (**A**), *groES* (**B**), *dnaK* (**C**), *dnaJ* (**D**) and *clpB* (**E**) relative to 16 S rRNA was quantified by qRT-PCR. Data are presented as the mean ± SEM of three independent experiments. Statistical significance was analyzed between PA and other groups by one-way ANOVA, Tukey t test (*p < 0.05; **p < 0.01, ***p < 0.001). (PA: *Pediococcus acidilactici*, PIN: phthalyl inulin nanoparticle, I: inulin, *groEL*, *groES*, *dnaK*, *dnaJ* and *clpB*: heat shock proteins, qRT-PCR: quantitative real-time polymerase chain reaction).

To analyze the patterns of gene expression alterations in PA with or without PIN4, high-throughput sequencing was performed to determine the mRNA expression levels. The sequencing results revealed several changes in the number of differentially expressed genes (DEGs) in PA/PINs. There were 930 DEGs among a total of 2,125 genes in the genome (*p* < 0.05). Among the DEGs, the expression levels of 31 genes were increased 1.5-fold in PA/PINs compared with PA, whereas the expression levels of 61 genes were decreased 1.5-fold in PA/PINs (Supplementary Fig. 7A,B). The genome of PA was annotated using rapid annotation subsystem technology (RAST), and the genes were categorized based on the variations in expression levels. As shown in the figure, the expression level of genes for RNA metabolism, DNA metabolism, and cell wall and capsule increased, whereas transcripts belonging to the carbohydrate category decreased (Supplementary Fig. 7C). Interestingly, the expression level of several genes (*groEL*, *groES*, *dnaK*, *dnaJ and clpB*) related to heat shock proteins of PA with internalized PINs increased significantly, suggesting that the internalization of PINs into probiotics led to changes in the expression of genes involved in the stress response (Supplementary Fig. 7D). Hence, the levels of gene expression related to the stress response, i.e., molecular chaperones (*groEL*, *groES and dnaK*), small molecular heat shock protein (*dnaJ*) and Clp protein (*clpB*), were further analyzed by qRT-PCR. Changes in the transcription level of *groEL*, *groES*, and *dnaK* in PA/PIN4 were statistically significant, showing 5.2, 4.6 and 4.4-fold increases, respectively, compared with PA alone (Fig. 6A–C). The transcription of *dnaJ* and *clpB* also increased 3.3 and 6.7-fold, respectively in PA/PIN4 compared with PA (Fig. 6D,E). Specifically, the expression levels of heat shock proteins in PA increased with the decrease in particle size of internalized PINs, suggesting that the greater the internalization of PINs into PA, the stronger is the induction of stress responses.

## Discussion

In this study, we developed PINs as a new formulation of prebiotics to enhance the antimicrobial potential of probiotics. PINs can be prepared by self-assembly of inulin conjugated to phthalic anhydride as hydrophobic groups to hydroxyl groups in inulin through hydrophobic interactions. Since primary alcohols are more reactive than secondary ones, it is assumed that the reaction occurs between the primary hydroxyl groups of the inulin and carboxylic acids of phthalic anhydride after ring opening through an esterification mechanism. Moreover, a higher conjugation rate of phthalic groups could result in smaller particle sizes of PINs, possibly due to the enhanced hydrophobic interactions between phthalic moieties.

Thus far, the study of the internalization of prebiotics into prokaryotes is still in an early stage, although a large amount of research has been focused on how foreign nanoparticles are internalized into eukaryotic cells through endocytosis^11^. Most research on prebiotic materials have mainly focused on fermentation by probiotics^12,13^. By contrast, one of the goals of our research is to develop prebiotics in nanoparticle form and to elucidate their internalization into probiotics. It has been reported that soluble prebiotics can enter probiotics by passive diffusion through transporters^14,15^. In contrast, the internalization of metal nanoparticles into *E*. *coli* has been reported to occur via electrostatic interactions^16^. However, we assumed that PINs would enter through transporters such as carbohydrate receptors at the cell surfaces of probiotics (PA). In support of our assumption, pretreatment with glucose significantly decreased the internalization rate of PIN4 into PA, while galactose and fructose inhibited the internalization to a reduced extent. These results revealed that the glucose units in the inulin backbone of PIN4 are preferably recognized by glucose transporters in PA to assist the internalization of PIN4.

Another goal of our study is to observe the effect of prebiotics on cellular and antimicrobial properties of probiotics. A number of studies have focused on the antimicrobial properties of metal nanoparticles against pathogens. These studies have revealed that the bactericidal ability was derived from the ionic interaction with the bacterial membrane^16^ or bacterial growth was abrogated by disrupting the membrane permeability^17^. However, metal nanoparticles have several limitations, such as aggregation and low stability *in vivo*. Although bacterial proteins have been used as capping agents to prevent the aggregation and stabilization of metal nanoparticles, these methods cause serious side effects in the host^18^. Another limitation is that metal nanoparticles inhibit both pathogens and beneficial microbes^19^. In contrast, the PINs used in this study did not show any toxicity toward PA, but rather they enhanced PA production and activity of pediocin. Although the mechanism is not clear, it seems that internalization of PINs by PA causes mild stress to activate the probiotic defense system, leading to increased production of pediocin. Hence, the treatment of PA with PINs greatly increased antimicrobial activity against both Gram-negative (SG) and Gram-positive (LM) pathogens compared with the treatment with inulin or PA itself. In particular, PA/PIN4 exhibited the highest antibacterial activity and pediocin production. These results indicated that the increased antibacterial activity of PA was dependent on the size and number of particles taken up by PA.

Probiotics are widely used in food and feed additives for their beneficial roles, such as immunomodulation, modulation of intestinal microflora, prevention of diarrhea^20^, and reduction of inflammation^20^. The benefits have been mostly focused on the antipathogenic activity of the bacteria, indicating that enhancing antimicrobial abilities tends to be central to probiotics research. Pediocin, a cationic peptide, is known as a strong antimicrobial peptide that is produced in *Pediococcus* species^21^. It is strongly active against LM^22^ and induces cell autolysis by forming a protein complex on cytoplasmic membranes^23^. The antimicrobial ability of pediocin has mostly been reported in Gram-positive pathogens, while pediocin can also inflict sublethal injuries in Gram-negative bacteria^23^. In our study, PIN treatment of PA markedly enhanced the production of pediocin. Consistent with this result, PA/PINs showed higher expression levels of *pedA* and *pedD* than other genes (Fig. 5G). We hypothesized that internalization of PINs could directly affect the production of pediocin because pediocin is a class 2a bacteriocin, and its production has been reported to represent a quorum-sensing phenomenon^24^. Pediocin is biosynthesized by four DNA fragments (*pedA*, *pedB*, *pedC* and *pedD*)^21^, and the induction factor is bacteriocin-like peptide with a double glycine leader without bacteriocin activity^25,26^. The *pedA* is called a prepediocin, which consists of a double-glycine leader sequence and has the same biological activity as bacteriocin^27,28^. The *pedD* gene encodes PedD, an ABC transporter that functions during pediocin secretion in cells. The proteolytic domain of PedD binds to prepediocin and removes the leader sequence to form mature pediocin. The removed double-glycine leader sequence from *pedA* can act as an induction factor^29^. Therefore, we consider the internalization of PINs to contribute to the higher antimicrobial ability of PA via expression of the *pedA* gene.

Notably, probiotics produce bacteriocin as the first defense system^29^, and therefore various factors such as cultural temperature^30^, pH, and pressure^31^ can affect the expression of bacteriocin by upregulating genes associated with the stress response, such as heat shock proteins (HSPs)^32^. Transcriptional analysis of the genes of PA with internalized PINs revealed significantly higher expression levels of HSPs (*groEL*, *groES*, and *dnaK*) than PA alone (Fig. 6 and Supplementary Fig. 7). The results indicated that internalization of PINs into PA caused a mild stress to induce the bacterial defense mechanism without cell death. Therefore, internalization of PINs into PA increased the expression of pediocin biosynthetic genes, modulated cell metabolisms, and activated the defense system. However, a comprehensive mechanistic study of the internalization of PINs into PA is required to unravel the mechanisms underlying the changes in expression levels of various genes in PA with internalized PINs.

In general, the production of lactic acid or H_2_O_2_ is closely related to the antimicrobial property of probiotics^33^. Interestingly, the internalization of PINs by PA did not affect the production of lactic acid (Fig. 5B) and H_2_O_2_ (Supplementary Fig. 6). Thus, it was clearly evident that the enhanced antimicrobial potential of PA with internalized PINs was largely due to pediocin production. In contrast, SCFA production was lower in PA/PINs than PA alone, but there were no significant differences among PA/PINs and PA. Transcriptional analysis revealed the different levels of gene expression in PA with internalized PINs (Supplementary Fig. 7). However, we could not find a direct relationship between the production of increased pediocin and decreased SCFAs at this time point. Further experiments are needed to verify the relationships between the internalization of PINs and the changes in cellular metabolism.

Ultimately, we can conclude that prebiotic nanoparticles can exert tremendous effects on probiotics leading to enhanced production of antimicrobial peptides that are effective against both Gram-positive and Gram-negative pathogens. Thus, our study highlights a novel strategy to produce antibacterial peptides in probiotics through intracellular stimulation by internalized inulin nanoparticles as a prebiotic, which holds great promise as a replacement for antibiotics in dairy, veterinary and human applications.

## Materials and Methods

### Materials

All the materials and chemicals used in this study were purchased from Sigma-Aldrich (St. Louis, MO, USA) unless otherwise stated. Lysogeny broth (LB), LB agar, De Man, Rogosa and Sharpe agar (MRS) broth, MacConkey agar and brain heart infusion (BHI) broth were purchased from BD Difco (Sparks, MD, USA) for bacterial cultures.

### Synthesis of phthalyl inulin nanoparticles (PINs)

PINs were synthesized according to a previously described method^34^ with a slight modification. Briefly, inulin (1 g, MW = 5000 gmol^−1^) was added to 5 ml of dimethyl formamide, and then 0.2 ml of 5% sodium acetate (w/v) was added as a catalyst for the reaction. Subsequently, phthalic anhydride was added to the inulin solution at various molar ratios, such as 0.3:1 (PIN1), 0.6:1 (PIN2), 1.2:1 (PIN3) and 2:1 (PIN4), to produce PINs with varying degrees of substitution of phthalate moieties in inulin. Four separate reactions were performed at 40 °C for 24 h under nitrogen. The produced PINs were dialyzed against distilled water at 4 °C for 24 h to form self-assembled nanoparticles of phthalic anhydride to inulin. Finally, the PINs were lyophilized and stored at −20 °C until use. Following the above protocol, acetyl inulin (AI) and propyl inulin (PrI) were synthesized, and similarly, the synthesized molar ratio was 1:1 between acetate anhydride:inulin and propionic anhydride:inulin.

### Characterization of PINs

The contents of the phthalyl group in PINs were confirmed by 600 MHz ^1^H-nuclear magnetic resonance (NMR) spectroscopy (AVANCE 600, Bruker, Germany). The surface topography of PINs was analyzed using a field-emission scanning electron microscope (FE-SEM) with SUPRA 55VP-SEM (Carl Zeiss, Oberkochen, Germany). PINs were mounted on the stubs with adhesive copper tape and coated with platinum under a vacuum using a coating chamber (CT 1500 HF, Oxford Instruments, Oxfordshire, UK). The sizes of the nanoparticles were measured with a dynamic light scattering (DLS) spectrophotometer (DLS-7000, Otsuka Electronics, Japan). The zeta potential of the nanoparticles was measured with an electrophoretic light scattering (ELS) spectrophotometer (ELS-8000, Otsuka Electronics, Japan).

### Tracking the internalization of PINs in probiotics

Initially, fluorescence isothiocyanate (FITC)-labeled PINs were prepared. Briefly, 5 mg of FITC was mixed with 100 mg of PINs or inulin dissolved in 1 ml of dimethyl sulfoxide (DMSO). After stirring for 4 h in dark at room temperature, the reaction mixture was dropped into 10 ml of ethanol to remove the unreacted FITC. FITC-PINs or FITC-inulin were collected by centrifugation at 19,000 × *g* for 10 min. The fluorescence of the FITC-PINs or FITC-inulin was then quantified using a standard curve of FITC-mannan. For the encapsulation of curcumin, 20 mg of curcumin was mixed with 100 mg of PIN4 dissolved in 1 ml of DMSO. The mixture was dialyzed against distilled water at 4 °C for 24 h to form self-assembled PIN4/curcumin particles, which were finally lyophilized and stored at −20 °C until use.

To observe the size-dependent internalization of PINs in probiotics, *Pediococcus acidilactici* KCTC 21088 (PA) (2.0 × 10^5^ CFU/ml) were inoculated into 1 ml of MRS broth, treated with 0.1% (w/v) FITC-PINs and incubated for 3 min at room temperature. After 3 min, the samples were washed with PBS and analyzed by flow cytometry and confocal laser microscopy (SP8 X STED, Leica, Wetzlar, Germany). To confirm the internalization of particles inside the probiotics, PA treated with FITC-PIN4 or PIN4/curcumin were analyzed by CLSM performed in Z-section mode.

To observe the temperature-dependent internalization of particles into probiotics, three separate cultures of PA were treated with 0.1% (w/v) FITC-PIN4 and incubated at 4 °C, 25 °C and 37 °C for 6 h. The samples were further washed with PBS and analyzed by flow cytometry and confocal laser microscopy. To observe the transporter-dependent internalization of particles into probiotics, glucose, galactose and fructose were used as blocking agents. PA (2.0 × 10^5^ CFU/ml) were inoculated into 1 ml of PBS and treated with 10% (w/v) glucose, galactose and fructose for 10 min at 37 °C before treatment with 0.1% (w/v) FITC-PIN4. After 6 h of incubation at 37 °C, the samples were washed three times with PBS, and the internalization of PIN4 was analyzed by flow cytometry and confocal laser microscopy.

### Observation of probiotics by field emission scanning electron microscopy (FESEM)

Samples for observation by SEM were prepared by following the method described by Zeitvogel *et al*.^35^. Briefly, pre-fixation was performed with Karnovsky’s fixation for 4 h, followed by three washes with 0.05 M sodium cacodylate buffer. Post-fixation was performed with 2% osmium tetroxide and 0.1 M cacodylate buffer for 2 h. After washing 2 times with distilled water, dehydration was performed using a series of graded ethanol solutions (30, 50, 70, 80, 90 and 100% ethanol in water). After dehydration, the samples were dried overnight using hexamethyldisilazane. Prior to SEM analysis, the samples were coated with Pt using an EM ACE200 (Leica, Austria) at 23 mA for 100 s and observed using an SEM (SUPRA 55VP, Carl Zeiss, Germany).

### Observation of probiotics by transmission electron microscopy (TEM)

Samples for observation by TEM were prepared by following the method described by Schrand *et al*.^36^. Fixation was performed using Karnovsky’s fixation for 4 h, followed by three washes with 0.05 M sodium cacodylate buffer and post-fixation with 2% osmium tetroxide and 0.1 M cacodylate buffer for 2 h. After washing two times with distilled water, the samples were dehydrated in a series of graded ethanol solutions (30, 50, 70, 80, 90 and 100% ethanol in water). The pellet was then incubated in 2 ml of propylene oxide and 1 ml of Spurr’s resin for 2 h, and then in 1 ml propylene oxide and 1 ml of Spurr’s resin overnight. Next, a fresh batch of 100% resin was added and cured at 60 °C for two days. After polymerization, the resin block was cut into 60–70-nm-thick sections using a Leica EM UC7 ultramicrotome. After staining with uranyl acetate, the samples were placed on 200 mesh copper grids, and images were obtained using a TEM (LIBRA 120, Carl Zeiss, Germany) operating at 120 kV.

### Bacterial cultures

All bacterial strains were cultured in the corresponding medium: *Pediococcus acidilactici* (PA) in MRS broth, Gram-negative *Salmonella* Gallinarum (SG) in LB broth, and Gram-positive *Listeria monocytogenes* (LM) in BHI broth. All bacterial cultures were incubated at 37 °C in a shaking incubator (255 rpm) for 24 h prior to being applied to the subsequent experiments or stored at −70 °C in 15% (v/v) glycerol.

### Co-culture and agar diffusion test for antimicrobial activity

Antimicrobial activities of PA against SG and LM strains were determined using the co-cultivation assay^37^ and agar diffusion test^38^ with some modifications. To quantitatively compare the antimicrobial activity of PA against SG by the co-cultivation assay, 2.0 × 10^5^ CFU/ml of SG was co-cultured with 2.0 × 10^5^ CFU/ml of PA treated with or without 0.5% (w/v) PINs or inulin in MRS broth for 8 h at 37 °C with aerobic condition in a shaking incubator (255 rpm). The degree of antimicrobial activity of PA against SG in the co-culture could be directly measured by the survival rate of SG. Hence, the co-culture samples were spread on MacConkey agar, incubated for 24 h at 37 °C and the number of SG colonies was counted. To test the antimicrobial activity of PA against LM using the co-cultivation assay, LM and PA were cultured in BHI broth and exposed to similar conditions as described above. Finally, the co-culture samples were spread on Oxford agar, and the number of LM colonies was counted.

Alternatively, the agar diffusion test was used to determine whether PA cultures treated with or without PINs were able to inhibit the growth of pathogens (SG or LM) on an agar plate *in vitro*. First, 120 µl of SG (2.0 × 10^8^ CFU/ml) was spread on an LB agar plate. A paper disc was placed on the SG-spread plate, and 120 µl (2.0 × 10^8^ CFU/ml) of PA culture treated with or without 0.5% (w/v) PINs or inulin was dropped onto the paper disc. After drying for 20 min at room temperature, the disc was cultured for 20 h at 37 °C. The zones of inhibition of SG growth, as a direct consequence of the antimicrobial activity of the PA cultures on the agar plate, were measured. Similarly, the same protocols were followed as above to observe the inhibitory effect on LM by PA cultures treated with or without 0.5% (w/v) PINs or inulin, excluding the tests performed on BHI agar plates.

To examine the concentration-dependent antimicrobial activity of probiotics, PA (2.0 × 10^5^ CFU/ml) were inoculated into 1 ml of MRS broth and treated with 0.5 or 1% (w/v) PINs or inulin. After 24 h of cultivation, the treated PA (2.0 × 10^5^ CFU/ml) were co-cultured with SG (2.0 × 10^5^ CFU/ml) in MRS broth for 8 h at 37 °C with shaking (255 rpm). The protocol for co-cultivation assay previously described was followed to quantitatively compare the antimicrobial activity of PA cultures toward SG. To examine whether the hydrophobic group was specifically required to enhance the antimicrobial ability, the antimicrobial activity of PA treated with acetyl inulin nanoparticles (AIN), propyl inulin nanoparticles (PrIN) was performed as described above.

### Analysis of growth condition and short chain fatty acid (SCFA) production by PA

PA were treated with or without PINs or inulin as described above. The growth conditions for the PA were monitored by measuring the pH and viable cell counts at the indicated time points. To detect the production of SCFAs by gas chromatography (GC), the cultured supernatants were mixed with an internal standard (propionic acid-2,2-d2) and methanol. GC analysis was conducted according to a method described by Arokiyaraj *et al*.^39^. GC was performed under the following conditions. The Thermo Scientific Trace 1310 system was used for GC, comprising a Thermo ISQ LT mass selective detector with a TG-5MS (Mass spectroscopy) column (30 × 0.25 mm (5%-phenyl)–methylpolysiloxane capillary column, film thickness of 0.25 lm). The temperature of the oven was programmed as follows: initial temperature of 50 °C for 5 min, then increases of 4 °C/min up to 250 °C. The carrier gas was helium, and the flow rate was 1.0 ml/min. Samples were injected in a volume of 1 µl, and the ionization energy was 70 eV. SCFAs were identified based on their retention time and by comparison of their mass spectral pattern with the National Institute of Standards and Technology library.

### Isolation, purification and analysis of pediocin

Pediocin was isolated and purified as described previously^40^ with some modifications. PA were treated with or without PINs or inulin as described above. The cultures were centrifuged at 3,000 × g for 30 min at 4 °C, and the supernatants were stirred with ammonium sulfate (35% v/v saturation) for 30 min. The precipitated proteins were obtained by centrifugation at 3,000 × g for 30 min at 4 °C. PBS buffer was added to dissolve the pellets, which were purified using a centrifugal filter from Sigma-Aldrich (St. Louis, MO, USA). The purified solutions were dialyzed against the buffer overnight. The dialyzed proteins were freeze-dried and stored at 4 °C for further analyses. While the protein concentration was determined by the Bradford assay, sodium dodecyl sulfate-polyacrylamide gel electrophoresis (SDS-PAGE) was used to observe and compare the isolated pediocin with a standard pediocin. Pediocin was quantified using a standard curve of bovine serum albumin.

The specific activity of pediocin was determined as previously described^41^ and was expressed as arbitrary units (AU) per ml. PA were treated with or without PINs or inulin as described above. Next, the culture supernatants were adjusted to pH 5.5 with 1 M sodium hydroxide to eliminate the antimicrobial effect of lactic acid. Pediocin activity was assayed by the agar well diffusion method and calculated based on the dilution ratio of the inhibitory activity. In brief, pediocin activity was determined by the diameter of the inhibition zone produced by PA (treated or untreated) that inhibited the growth of LM.

### Quantitative real-time PCR

RNA extraction was performed using the TRIzol® Max™ Bacterial RNA Isolation Kit purchased from Thermo Fisher Scientific Inc. (Waltham, MA, USA). Total RNA extraction was conducted according to the manufacturer’s instructions. Briefly, PA were treated with or without PINs or inulin as described above. After the isolation of RNA, cDNA was synthesized from 1 µg of RNA using ReverTra Ace® qPCR RT Master Mix with gDNA Remover purchased from TOYOBO CO., LTD (Dojima, Osaka, Japan). Quantitative real-time PCR (qRT-PCR) was performed with SYBR qPCR Mix using one-step real-time PCR. The primer sequences are listed in Table. S1 were designed based on the sequence reported by Fernandez *et al*.^42^. For relative quantification, 0.01 ng of 16 s rRNA cDNA was used when 1 ng of the *pedA*, *pedB*, *pedC* and *pedD* genes was used. The relative gene expression was calculated using the ΔΔCt method. The target gene expression was normalized to the relative expression of 16 s rRNA as an internal control in each sample. The data are presented as the relative fold-change compared with the probiotics control group.

### Sequencing and analysis of mRNA

For high-throughput sequencing, RNA was extracted at 24 h after treatment of PA with PIN4, and sequencing libraries were constructed using the TruSeq RNA kit (Illumina, CA, USA) according to the manufacturer’s instruction. The prepared libraries were then sequenced using HiSeq. 2500 (Illumina, CA, USA) for 100-bp paired-end reads. Adapter sequences of the reads were trimmed with Cutadapt1.10^43^, and ribosomal RNA sequences were removed in silico using the SortMeRNA program^44^. The sequence reads were quality-filtered using in-house Perl scripts^45^. In brief, when 95% of the nucleotide bases in a read were given a quality score over 31 (Illumina 1.8+) and the read length was ≥70 bp, the read was used for transcript analysis. RNA-seq reads were mapped to the PA genome (NCBI accession MPJU00000000) using TopHat^46^, and HTSeq was used to quantify the gene expression^47^. EdgeR was used to quantify and normalize the gene expression^47^. All programs were used with default options, and the gene expression level was normalized by fragments per kilobase of transcript per million fragments sequenced (FPKM). The genome of PA was annotated using rapid annotation subsystem technology (RAST) with default options, and all genes were categorized by this technology^48^. The mRNA sequences were registered in the NCBI Sequence Read Archive under accession SRR5411014.

For RNA extraction and quantification, the above-described protocols were followed. qRT-PCR was performed with SYBR qPCR Mix using one-step real-time PCR. All primers were designed using primer 3 software, and their sequences from 5′ to 3′ are shown in Table. S1.

### Hydrogen peroxide activity assay

PA (2.0 × 10^5^ CFU/ml) was inoculated into 1 ml of MRS broth and treated with 0.5% (w/v) of PINs or inulin. After incubation for 24 h at 37 °C with shaking (246 rpm), the pH of the culture supernatant was adjusted to 6. The culture supernatant was treated with 1 mg/ml catalase and incubated at 37 °C for 2 h. After incubation, the supernatant was heated at 100 °C for 30 min. Next, 5.0 × 10^7^ CFU/ml of SG were treated with 1 ml of the culture supernatant and incubated at 37 °C for 6 h with shaking (246 rpm). The SG CFU for each culture supernatant were measured using MacConkey agar.

### Accession number

The RNA-seq data have been deposited in the NCBI Sequence Read Archive under accession SRR5411014.

## Electronic supplementary material

Supplementary Information
