# Supplementary material for: A new way of producing pediocin in Pediococcus acidilactici through intracellular stimulation by internalized inulin nanoparticles
Source: Sci Rep. 2018 Apr 12;8:5878. doi: 10.1038/s41598-018-24227-z (PMC5897564; doi:10.1038/s41598-018-24227-z)
Supplement: Supplementary file 1 — Supplementary Information [file 41598_2018_24227_MOESM1_ESM.pdf]

A new way of producing pediocin in *Pediococcus acidilactici* through intracellular stimulation by internalized inulin nanoparticles

Whee-Soo Kim[1+], Jun-Yeong Lee[1+], Bijay Singh[2,3], Sushila Maharjan[1,3], Liang Hong[1], Sang-Mok Lee[1], LianHua Cui[5], Ki-June Lee[1], GiRak Kim[1], Cheol-Heui Yun[1,2], Sang-Kee Kang[4], Yun-Jaie Choi[1,2]* and Chong-Su Cho[1,2]*

[1]Department of Agricultural Biotechnology, Seoul National University, Seoul 08826, Republic of Korea.

[2]Research Institute of Agriculture and Life Science, Seoul National University, Seoul 08826, Republic of Korea

[3]Research Institute for Bioscience and Biotechnology, Kathmandu 44600, Nepal

[4]Institute of Green-Bio Science & Technology, Seoul National University, Pyeongchang, Gangwon-do 25354, Republic of Korea

[5]Co-Innovation Center of Beef Cattle Science and Industry Technology, Yanbian University, Yanji, Jilin 133002, P. R. China

[+]W.S. Kim and J.Y. Lee are equally contributed.

[*] Corresponding authors with equal contribution: Yun-Jaie Choi (cyjcow@snu.ac.kr) and Chong-Su Cho (chocs@snu.ac.kr)

Whee-Soo Kim: gnltngnltn@snu.ac.kr

Jun-Yeong Lee: akirus86@snu.ac.kr

Bijay Singh: singhbijay@hotmail.com

Sushila Maharjan: ruma_sushila@hotmail.com

Liang Hong: hl0626@snu.ac.kr

Sang-Mok Lee: allees@snu.ac.kr

LianHua Cui: cuilianhua1980@naver.com

Ki-June Lee: zenith09@snu.ac.kr

GiRak Kim: enxrak1@snu.ac.kr

Cheol-Heui Yun: cyun@snu.ac.kr

Sang-Kee Kang: kangsk01@snu.ac.kr

Yun-Jaie Choi: cyjcow@snu.ac.kr

Chong-Su Cho: chocs@snu.ac.kr

Supplementary Information

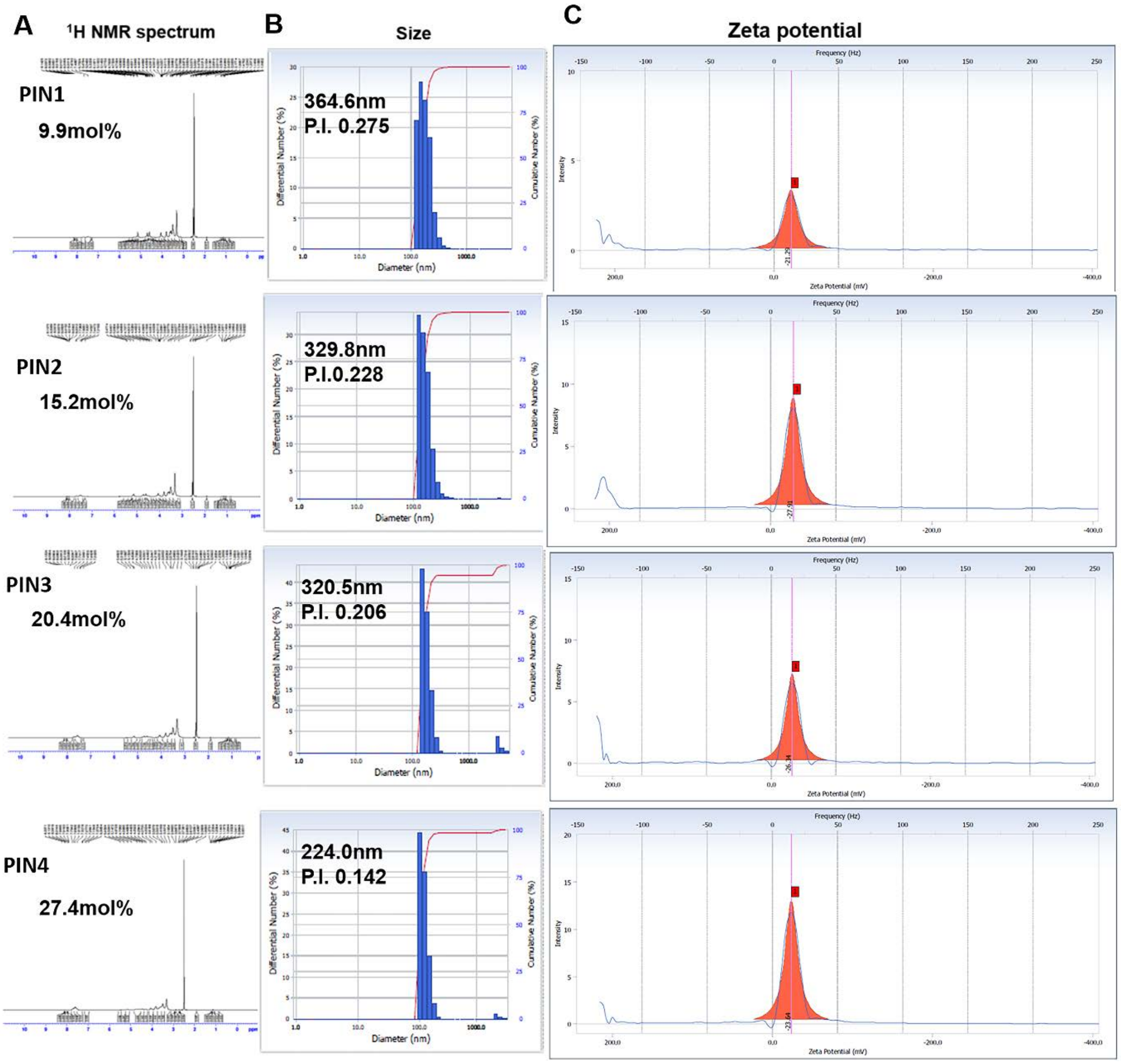

Supplementary Figure 1. Characteristics of PINs. Calculation of mol.-% of phthalic acid in PINs by 1H-NMR spectroscopy (A). Measurement of the sizes of PINs by DLS (B) and zeta-potential by ELS (C). (PIN: phthalyl inulin nanoparticle, H-NMR: nuclear magnetic resonance, DLS: dynamic light scattering, ELS: electrophoretic light scattering).

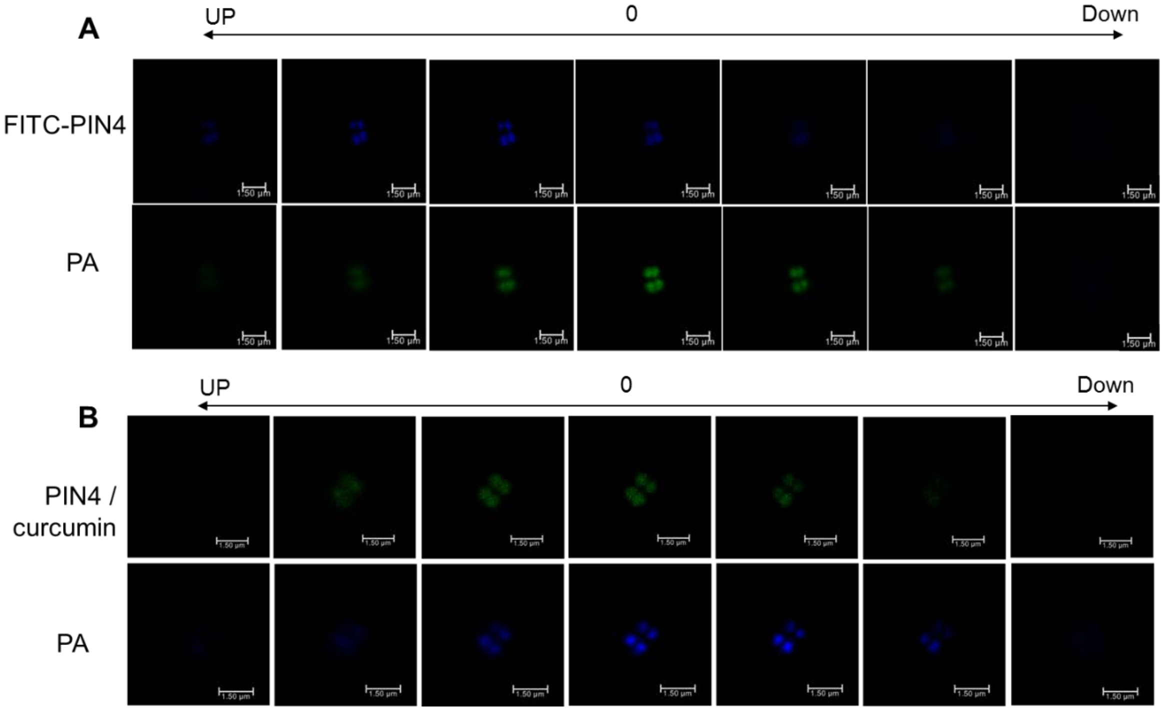

Supplementary Figure 2. Analysis of the internalization of PINs into PA. Confocal images were obtained after treatment of PA with FITC-PIN4 (A) and PIN4/curcumin (B) for 3 min at room temperature. Z-section images show the internalization of corresponding PINs into PA. FITC-PIN4 or PIN4/curcumin is shown in green, and PA was stained blue with DAPI. Scale bar=1.5 μm (A, B). (PA: *Pediococcus acidilactici*, PIN: phthalyl inulin nanoparticle, FITC: fluorescein isothiocyanate, DAPI: 4',6-diamidino-2-phenylindole).

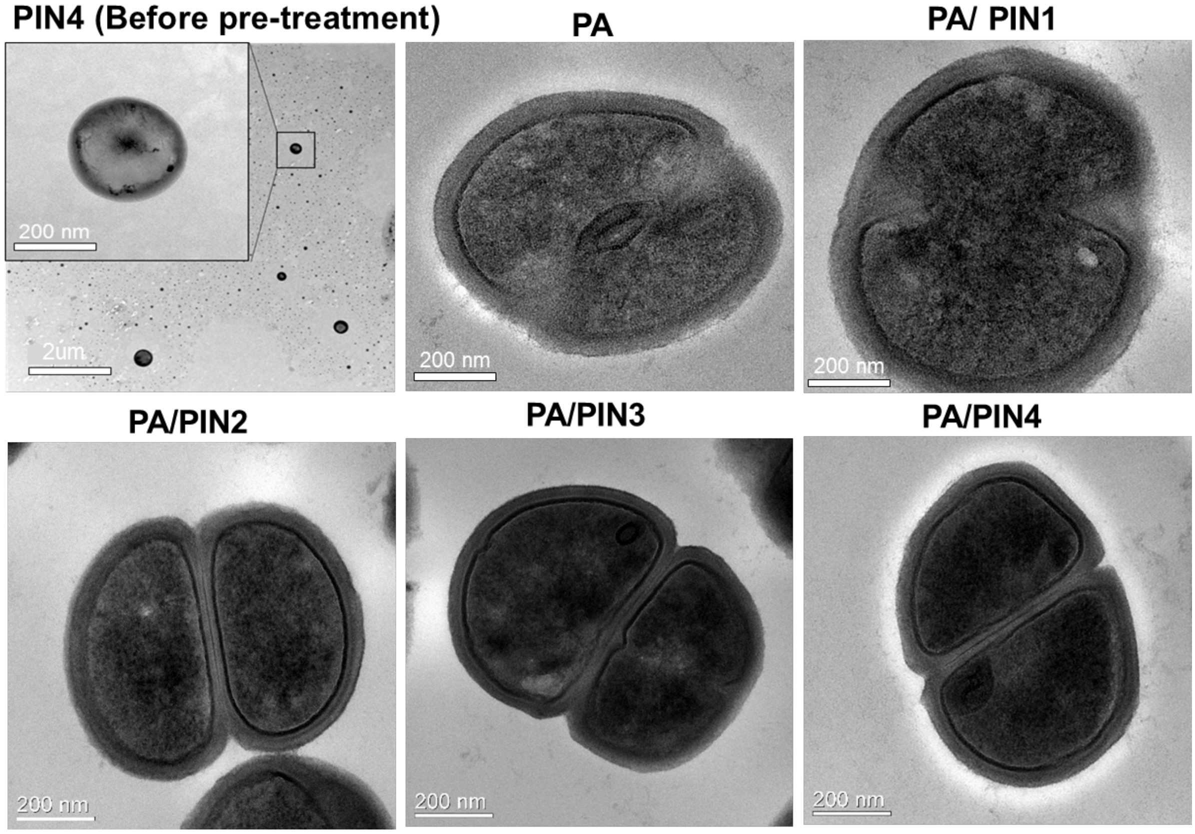

Supplementary Figure 3. Analysis of the internalization of PINs by TEM. PA were treated with PINs for 24 h and analyzed by TEM to visualize the internalization of PINs into the probiotics. (PA: *Pediococcus acidilactici*, PIN: phthalyl inulin nanoparticle, TEM: transmission electron microscopy).

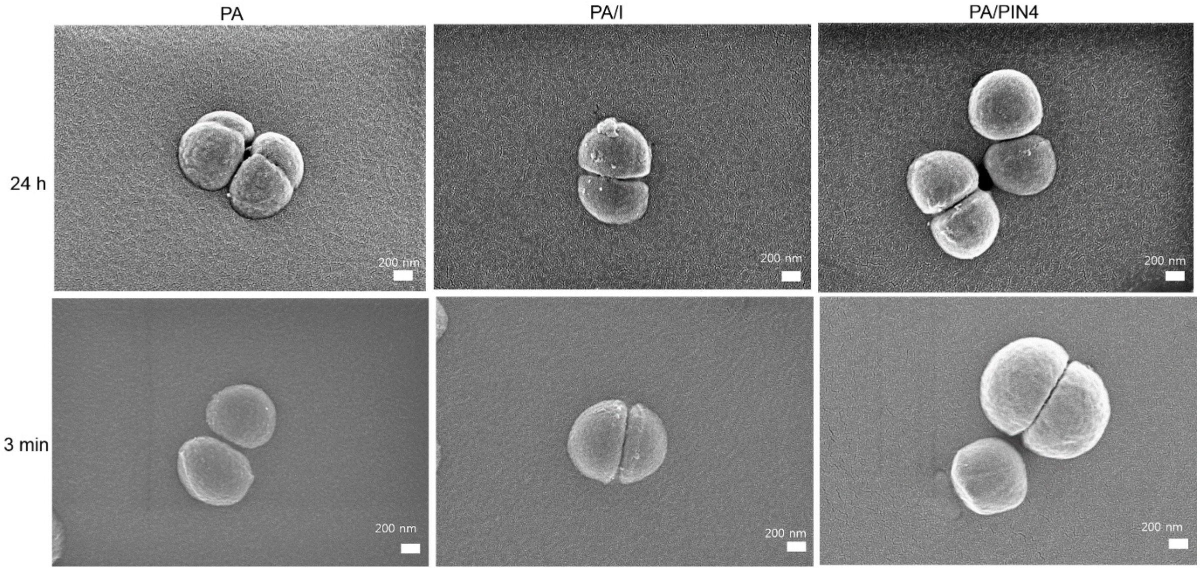

Supplementary Figure 4. Morphology of PA with or without treatment. PA were treated with inulin (I) or PIN4 for 3 min or 24 h and analyzed by SEM to visualize any structural changes in PA. (PA: *Pediococcus acidilactici*, PIN: phthalyl inulin nanoparticle, SEM: scanning electron microscope).

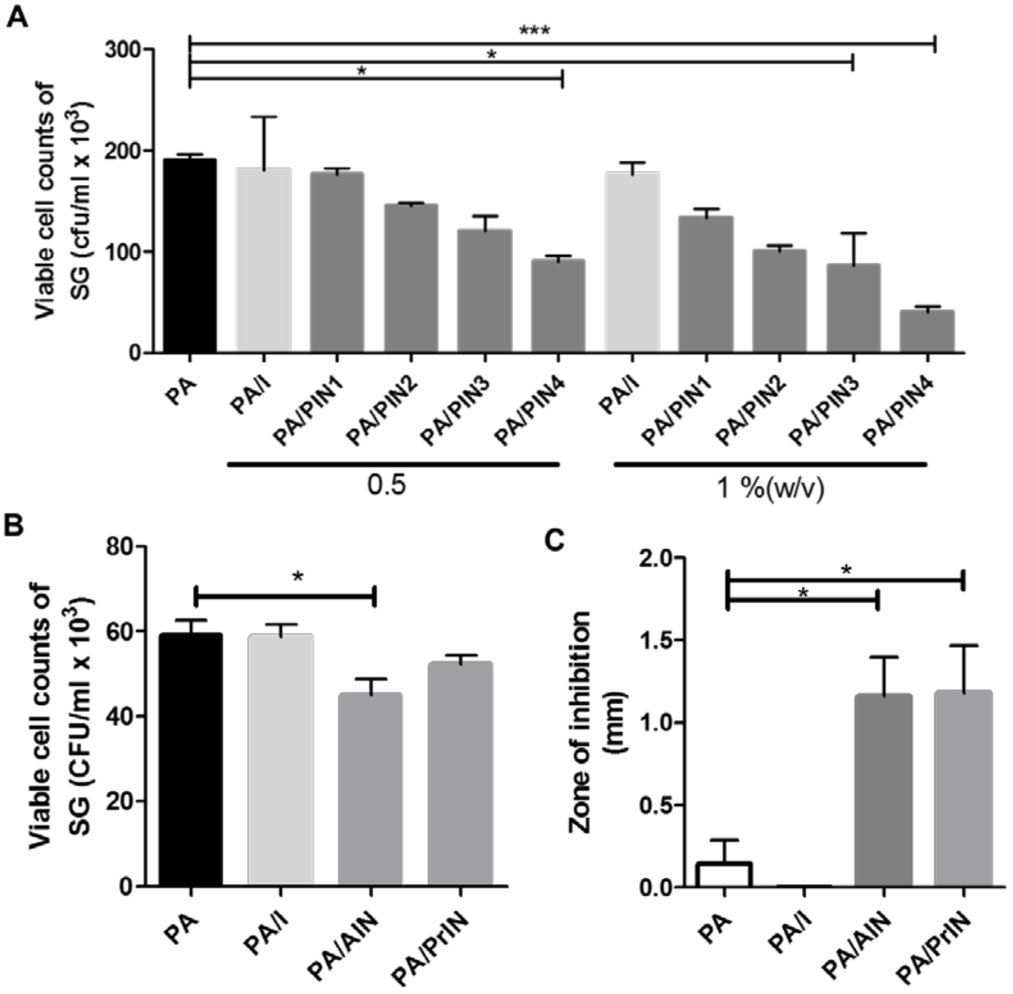

Supplementary Figure 5. Antimicrobial activity of PA against SG. PA treated with PINs or inulin were cultured with Gram-negative SG, and the growth inhibition was calculated by CFU for SG **(A)**. Antimicrobial activity of PA after treatment with or without AIN and PrIN against SG **(B)**. The diameters of the growth inhibition of SG by PA treated with or without AINs and PrINs on LB agar plates **(C)**. Data are presented as the mean ± SEM of three independent experiments. Statistical significance was analyzed by one-way ANOVA, Tukey t test (*$p<0.05$, **$p<0.01$, ***$p<0.001$). (PIN: phthalyl inulin nanoparticle, AIN: acetyl inulin nanoparticle, PrIN: propyl inulin nanoparticle, I: inulin, CFU: colony forming unit, PA: *Pediococcus acidilactici*, SG: *Salmonella* Gallinarum, LB: lysogeny broth).

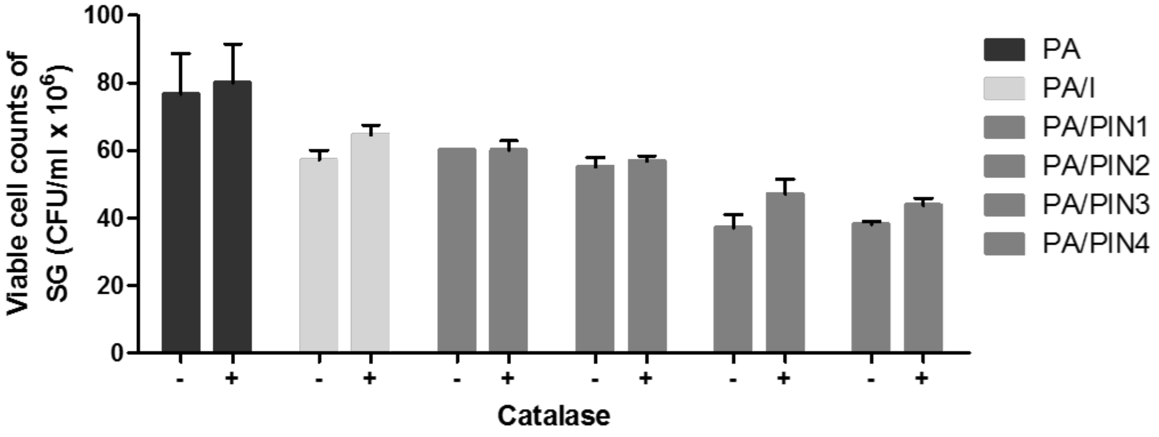

Supplementary Figure 6. Hydrogen peroxide activity assay. Growth of SG culture supernatants: +: catalase treatment, -: no catalase treatment. Each experiment was performed in triplicate, and each point represents the relative mean value. Data are presented as the mean ± SEM of three independent experiments. Statistical significance was analyzed by one-way ANOVA, Tukey t test (*p<0.05, **p<0.01, ***p<0.001). (PA: *Pediococcus acidilactici*, PIN: phthalyl inulin nanoparticle, I: inulin, SG: *Salmonella* Gallinarum).

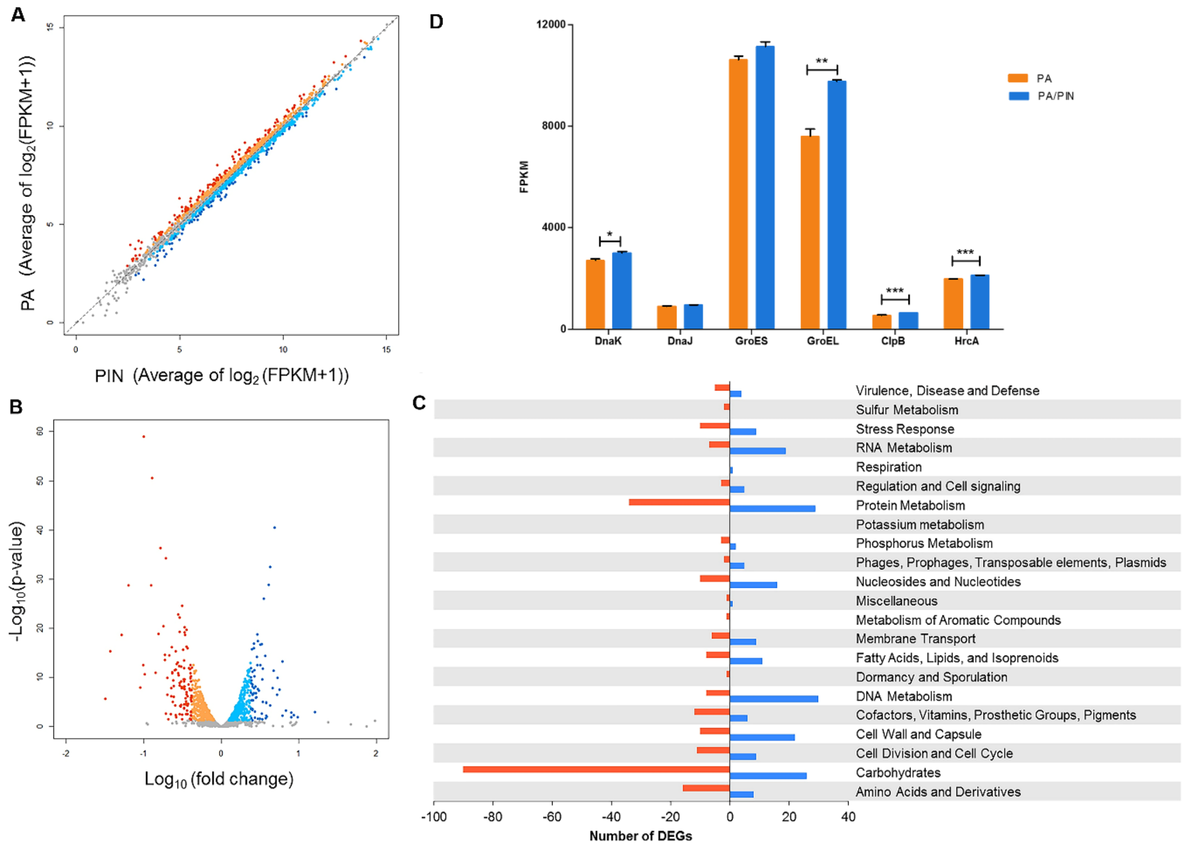

Supplementary Figure 7. Distribution of differentially expressed genes (DEGs) between PA and PA with internalized PIN4 ($p < 0.05$). Scatter plot representing the distribution and expression levels of the examined genes **(A)** and volcano plot representing the statistical significance with respect to PIN4 **(B)** are presented. Red dot, PA-preferential gene (fold change ≥ 1.5); yellow dot, PA-preferential gene (fold change < 1.5); blue dot, PIN-preferential gene (fold change ≥ 1.5); azure dot, PIN-preferential gene (fold change < 1.5); gray dot, non-DEG. (C) Functional categorization of the DEGs with RAST. **(D)** Gene expression related to stress response. Data are presented as the mean ± SEM of three replicates. Statistical significance was analyzed by one-way ANOVA, Tukey t test (*p<0.05, **p<0.01, ***p<0.001). (PA: *Pediococcus acidilactici*, PIN: phthalyl inulin nanoparticle, RAST: *rapid annotation* using subsystem technology, FPKM: fragments per kilobase million).

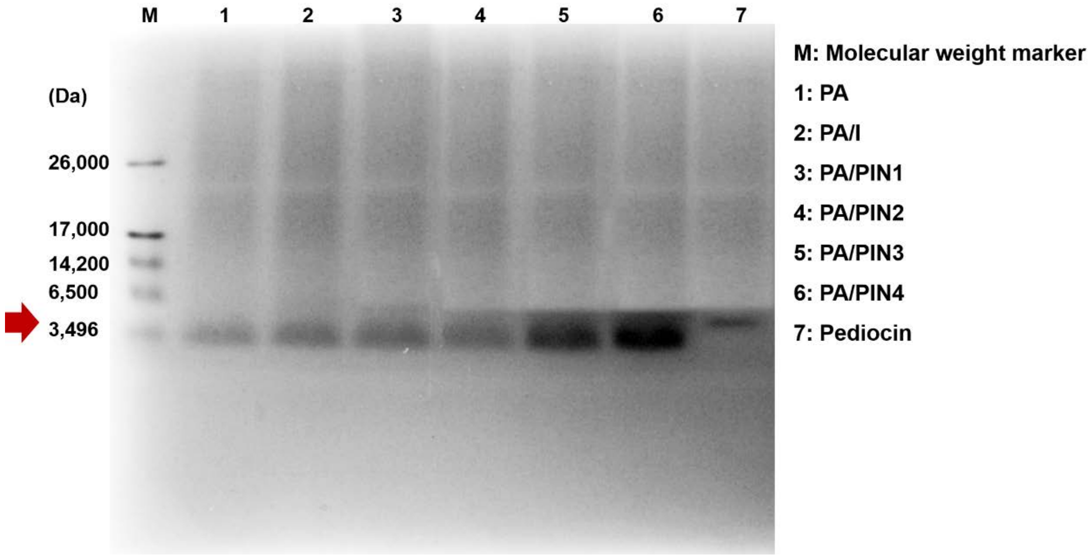

Supplementary Figure 8. SDS-PAGE of pediocin. The molecular weight of pediocin was determined by SDS-PAGE using a reference pediocin. Isolation of pediocin were performed after treatment of PA with 0.5 % (w/v) PINs or I for 24 h at 37 °C. (PA: *Pediococcus acidilactici*, PIN: phthalyl inulin nanoparticle, I: inulin, SDS-PAGE: sodium dodecyl sulfate polyacrylamide gel electrophoresis).

Supplementary Table 1. List of the primers used in this study

	Primer sequence (5'-3')	Size (bp)	
ped A	f:TGGCAAACATTCCTGCTCTGT	83	Structure protein
	r:CACCAGTAGCCCATGCCATAG		
ped B	f:ATTGCCAGCCAAGCGTTAGT	102	Immunity protein
	r:GCCCCACCCTTTTTGAGAAT		
ped C	f:CCATATCGGTGAG TGCTGACA	104	ABC transporter
	r:AGGAATAACGCCCCTGATGTT		
ped D	f:GGCCCATCTTCGACAGCTT	101	Regulatory protein
	r:GCACAGCTTCGGCATTTAAAT		
16S	f:GATGCGTAGCCG ACCTGAGA	113	
	r:TCCATCAGACTTGCGTCCATT		
dnaK	f:TTAACACGGGCACAATTTGA	212	
	r: GCTTCGTCAGGGTTAATGGA		
dnaJ	f: GCCCAACTTGTGGTGGTACT	240	
	r:CCAGTGCAGCTTGTACGAAA		
groEL	f:GGTAACGGTCGCGTTTTAGA	156	
	r:TTCAACGACTGCAACTAAGTCC		
groES	f:GGAAGACCTTGACGCAGAAG	239	
	r:CGTTTTGAAGTGCTGAACGA		
clpB	f:CGGCAGCCAAGTTATCTAGC	219	
	r:GCAGTGCCTTTAAGCGTTTC		
